# DNA prime-protein boost based vaccination with a conserved region of leptospiral immunoglobulin-like A and B proteins enhances protection against leptospirosis

**DOI:** 10.1590/0074-02760150222

**Published:** 2015-12

**Authors:** Karine M Forster, Daiane D Hartwig, Thaís L Oliveira, Kátia L Bacelo, Rodrigo Schuch, Marta G Amaral, Odir A Dellagostin

**Affiliations:** 1 Universidade Federal de Pelotas, Centro de Desenvolvimento Tecnológico, Núcleo de Biotecnologia, Programa de Pós-Graduação em Biotecnologia, Pelotas, RS, Brasil; 2 Universidade Federal de Pelotas, Instituto de Biologia, Departamento de Microbiologia e Parasitologia, Pelotas, RS, Brasil

**Keywords:** leptospirosis, Leptospira, LigBrep, subunit vaccine, DNA vaccine, prime-boost

## Abstract

Leptospirosis is a zoonotic disease caused by pathogenic spirochetes of the*Leptospira* genus. Vaccination with bacterins has severe limitations. Here, we evaluated the N-terminal region of the leptospiral immunoglobulin-like B protein (LigBrep) as a vaccine candidate against leptospirosis using immunisation strategies based on DNA prime-protein boost, DNA vaccine, and subunit vaccine. Upon challenge with a virulent strain of*Leptospira interrogans*, the prime-boost and DNA vaccine approaches induced significant protection in hamsters, as well as a specific IgG antibody response and sterilising immunity. Although vaccination with recombinant fragment of LigBrep also produced a strong antibody response, it was not immunoprotective. These results highlight the potential of LigBrep as a candidate antigen for an effective vaccine against leptospirosis and emphasise the use of the DNA prime-protein boost as an important strategy for vaccine development.

Leptospirosis, an emerging zoonotic disease determined by pathogenic species of*Leptospira*, constitutes a major public health problem worldwide (Adler & Moctezuma 2010). Pathogenic leptospires colonise the host proximal renal tubules, which allow their dissemination to the environment *via* urine. Fever, chills, headache, and severe myalgia characterise the early phase of disease. Approximately 10% of infected patients develop a severe illness with multiorgan system complications, including hepatic dysfunction with jaundice, acute renal failure, pulmonary haemorrhage, and acute respiratory distress, presenting high mortality rates (50-70%) (Segura et al. 2005, Gouveia et al. 2008, Hartskeerl et al. 2011).

Vaccination with inactivated whole-cell preparations (bacterins) fail to afford long and cross-protective immunity against different *Leptospira* serovars (Koizumi & Watanabe 2005). Several studies have shown the potential of the *Leptospira* surface antigens as vaccine candidates in experimental animal models (Dellagostin et al. 2011). The genes encoding the leptospiral immunoglobulin-like (Lig) proteins are upregulated at physiological osmolarity (Matsunaga et al. 2005) and encode surface-exposed proteins (Matsunaga et al. 2003), which bind extracellular matrix proteins (Choy et al. 2007, Lin & Chang 2008, Lin et al. 2009) and human complement regulators (Castiblanco-Valencia et al. 2012, Choy 2012), possibly contributing to host-pathogen interactions. Regarding the genetic diversity of *lig* genes,*ligB* is present in all pathogenic *Leptospira* spp, unlike *ligA* (McBride et al. 2009). The heterologous expression of pathogen-specific genes*ligA* and *ligB* in the saprophyte *Leptospira biflexa* results in a virulence-associated phenotype and enhanced adhesion to cultured cells and fibronectin (Figueira et al. 2011).

The protective efficacy of Lig proteins as subunit vaccines has already been demonstrated in hamsters; however, no sterilising immunity was observed (Silva et al. 2007). In a recent study, we demonstrated that the portion shared by the LigA and leptospiral immunoglobulin-like B protein (LigBrep) used as a DNA vaccine is a potential vaccine candidate, affording partial protection against heterologous challenge (Forster et al. 2013a). Together, these data suggest that LigBrep is a potential candidate antigen for the development of a vaccine against leptospirosis.

Genetic immunisation is able to induce humoral and cellular immunity, persistent expression of heterologous antigen, and a memory response against the infectious disease. Despite these advantages, the major limitation of DNA immunisation is its poor immunogenicity (Babiuk et al. 2000). The DNA prime-protein boost strategy, in which the immune response is primed with a DNA vaccine and subsequently boosted with a protein or vector (e.g., viruses or bacteria), expressing the antigen, constitutes a promising approach to improve the efficiency of DNA immunisation (Feng et al. 2009, Lu 2009, Hartwig et al. 2013).

In the present study we immunised hamsters by DNA, protein, or prime-boost based vaccination, using LigBrep as antigen and Alhydrogel as adjuvant, and determined the efficacy of these vaccination strategies in eliciting an IgG antibody response and in affording protective and sterilising immunity against heterologous challenge in hamsters.

## MATERIALS AND METHODS

*Bacterial strains and culture conditions - Leptospira interrogans*serovar Canicola strain Hond Utrecht (HU) IV and *L. interrogans*serovar Copenhageni strain Spool (Forster et al. 2013b) were grown in Ellinghausen-McCullough-Johnson-Harris (EMJH) medium (Difco, BD Diagnostics, USA) supplemented with *Leptospira*Enrichment EMJH (Difco) at 30ºC. *Escherichia coli* strain TOP10 (Invitrogen, USA) was cultivated in Luria-Bertani (LB) medium at 37ºC with the addition of ampicillin to 100 µg.mL^-1^.

*Vaccine construction* - The DNA sequence corresponding to the*ligBrep* fragment (1-1,884 bp) was amplified by polymerase chain reaction (PCR) from the *L. interrogans* serovar Canicola strain HU genome using oligonucleotides designed according to the genome sequence of*L. interrogans* serovar Copenhageni strain Fiocruz L1-130 (GenBank accession AE016823). Then, it was cloned into pTARGET (Promega, USA) and pAE vectors (Ramos et al. 2004) for use as DNA and subunit vaccines, respectively, as described (Forster et al. 2013a). Briefly, for DNA vaccine construction, the fragment amplified by PCR was cloned into the pTARGET^TM^ mammalian expression vector. *E. coli* TOP10 electrocompetent cells were transformed with the recombinant vector and cultured in LB medium at 37ºC.

DNA was extracted with the Plasmid DNA Purification Nucleo Bond Xtra Maxi kit (Macherey-Nagel, Germany) and quantified with a Qubit Fluorometer (Invitrogen). The DNA vaccine functionality was evaluated in VERO cells transfected with the plasmid pTARGET/*ligB*rep, using the transfection reagent Lipofectin (Invitrogen) as previously described (Forster et al. 2013a). Recombinant fragment of LigBrep (rLigBrep) LigBrep expression was observed by indirect immunofluorescence and the reading was obtained with a fluorescence microscope at 400X magnification.

The 6x His-tagged recombinant LigBrep protein region [1-628 amino acids (aa)] was expressed in *E. coli* BL21 (DE3) Star cells, solubilised using 8 M urea and purified by immobilised metal ion affinity chromatography using Ni2 Sepharose HisTrap columns. Fractions containing eluted protein were visualised by sodium dodecyl sulfate polyacrylamide gel electrophoresis and Western blot. Dialysis was performed at 4ºC in phosphate-buffered saline (PBS) containing decreasing concentrations of urea in each step. The protein was quantified using the BCA Protein Assay Kit (Pierce, USA), with bovine serum albumin as the standard.

*Animals* - Female Golden Syrian hamsters were housed at the animal facility of the Federal University of Pelotas (UFPel). All the animal experiments were approved by the Committee on the Ethics of Animal Experiments of the UFPel. The animals were maintained in accordance with international guidelines throughout the experimental period.

*Immunisations and challenge experiment* – Four-five-week-old female Golden Syrian hamsters were allocated into groups of five or six animals, and food and water were provided *ad libitum*. Animals were inoculated into the quadriceps muscle on days 0 and 21 with either rLigBrep (100 µg) or pTARGET/*ligBrep* (100 µg) plus 15% Alhydrogel adjuvant, as follows: pTARGET/*ligBrep* + rLigBrep (DNA prime-protein boost), pTARGET/*ligBrep* + pTARGET/*ligBrep* (DNA/DNA), and rLigBrep + rLigBrep (protein/protein). The negative control group was inoculated with empty pTARGET plasmid (100 µg) or PBS + 15% Alhydrogel. Additionally, a separate group was inoculated with pTARGET/*ligBrep* + pTARGET/*ligBrep* (DNA/DNA) without adjuvant. The positive control group was immunised with 109 heat-killed whole-leptospires (bacterin). Forty-two days after the first dose was administered, all hamsters were challenged intraperitoneally with 10 leptospires, equivalent to 5x median lethal dose of the*L. interrogans* strain Spool (Forster et al. 2013b). Blood samples were collected from the retroorbital plexus before each immunisation and challenge, and the sera were stored at -20ºC. Hamsters were monitored daily for morbidity and euthanized when clinical signs of terminal disease appeared, such as loss of appetite, gait difficulty, dyspnoea, prostration, ruffled fur, or weight loss of ≥ 10% of the animal’s maximum weight.

*Humoral immune response* - Antibody responses were monitored by ELISA. Briefly, ELISA plates (Polysorp Surface, Nunc; Thermo Scientific, USA) were coated for 16-18 h at 4ºC with 200 ng of rLigBrep added per well, diluted in carbonate-bicarbonate buffer (pH 9.6). The plates were washed three times with PBS with 0.05% [v/v] Tween 20 (PBST) and blocked. Hamster sera were added at a 1:50 dilution for 1 h at 37ºC, followed by three washes with PBST. Goat anti-hamster IgG peroxidase conjugate (1:6,000 dilution; Serotec, UK) was added and incubated at 37ºC for 1 h and washed five times with PBST. The reaction was visualised with*o*-phenylenediaminedihydrochloride (Sigma-Aldrich, Brazil). The reaction was stopped by the addition of 0.1 M sulphuric acid and absorbance was determined at 492 nm using a Multiskan MCC/340 ELISA reader (Titertek Instruments, USA). Mean values were calculated from sera samples assayed in triplicate.

*Culture and histopathology assay* - Surviving hamsters were euthanized on day 30 post challenge, and kidney and lung tissues were collected for histopathology and culture. Kidney samples were used to confirm sterilising immunity by culture in EMJH medium (pH 7.2). Dark-field microscopy was performed during an eight-week incubation period to identify positive cultures. For histopathological studies, kidney and lung tissues samples were fixed in 10% formalin (pH 7.0) and embedded in paraffin. Six sections of 5-6 µm thickness from each organ were stained with haematoxylin and eosin and examined by a qualified pathologist.

*Imprint detection* - The presence of leptospires in the kidneys of immunised hamsters was evaluated by the imprint method (Chagas-Junior et al. 2009). Briefly, imprints were obtained by direct pressure of the cut surface of the tissue sample onto poly-ʟ-lysine-coated glass slides. Imprint slides were dried at room temperature, fixed in methanol for 10 min at 4ºC, and incubated for 30 min in a dark humid chamber at 30ºC. After three washes with 10% (v/v) foetal bovine serum (FBS) diluted in PBS, anti-LipL32 mAb (1D9) diluted 1:100 was added and the imprints were incubated in a dark humid chamber at 30ºC for 1 h. Next, the imprints were incubated for 1 h under the same conditions with an anti-*Leptospira* fluorescein isothiocyanate conjugate, diluted 1:100, after three washes with PBS plus FBS. Nucleic acids were visualised by counterstaining with Hoechst dye (diluted 1:10) for 30 min at 30ºC in a dark, humidified chamber. Following five washes with PBS plus FBS, mounting medium was added and a cover slip was sealed in place with acrylic. Staining was visualised by fluorescence microscopy (Olympus, Japan) at an excitation wavelength of 450 nm.

*Statistical analysis* - Variance analysis was used to determine significant differences between the assay results. The Bonferroni test was used to determine significant differences in serological assays. The Fisher exact test and the Wilcoxon log-rank test were used to determine significant differences for mortality and survival, respectively, using Prism 5 (Graphpad, USA). Differences were considered significant at p < 0.05.

## RESULTS

*Vaccine preparation* - The expression of LigBrep in the pTARGET/*ligBrep* construct was confirmed by detection of rLigBrep in transfected VERO cells using polyclonal anti-LigB sera. No reaction was detected in VERO cells transfected with the empty pTARGET plasmid. The rLigBrep protein region was expressed by *E. coli* BL21 (DE3) in insoluble inclusion bodies. The protocol using urea for solubilisation was efficient, resulting in a yield of approximately 5 mg·L^-1^. After refolding, sera of animals naturally infected with virulent leptospires recognised the recombinant protein in a dot blot assay under native conditions (data not show), showing that the antigenicity was not affected by the solubilisation process.

**Fig. 1:**
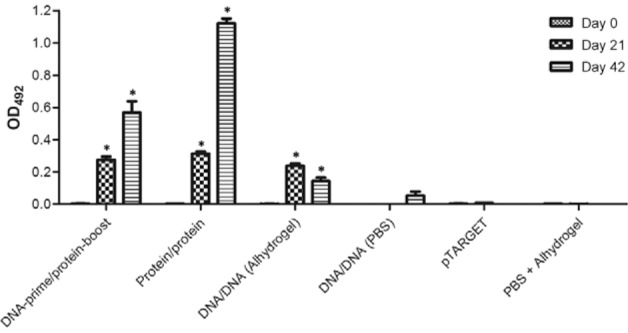
: humoral immune response in hamsters immunised with LigBrep vaccines measured by ELISA. Recombinant protein LigBrep was used as antigen. Results are expressed as the mean absorbance of all animals in each group. Asterisk means p < 0.05 in comparison to the negatives control group, which received the empty pTARGET plasmid or phosphate-buffered saline (PBS) + Alhydrogel. OD: optical density.

*Antibody response in LigBrep-immunised hamsters* - The IgG-specific response induced by vaccines was evaluated at different time points (0, 21, and 42 days) by ELISA. The results shown in Fig. 1revealed that significant antibody levels were induced by all vaccination strategies. Hamsters immunised with LigBrep-based vaccines presented higher IgG levels than control groups at 21 days post-inoculation (DPI) (p < 0.05) and these levels increased at 42 DPI. As shown in Fig. 1, significant levels of specific antibodies were not detected in the negative control groups (pTARGET or PBS + Alhydrogel) or in the hamsters that received the DNA/DNA vaccine without Alhydrogel as adjuvant in the formulation.

**Fig. 2:**
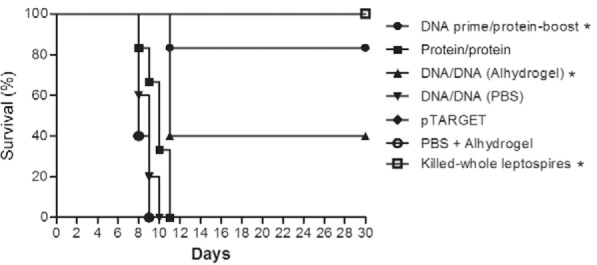
: survival curve of hamsters immunised with LigBrep vaccines or with killed-whole leptospires after heterologous challenge with 101 virulent Leptospira interrogans strain Spool. The Wilcoxon log-rank test was used to determine significant survival differences between immunised groups and the controls [empty pTARGET plasmid or phosphate-buffered saline (PBS) + Alhydrogel]. p < 0.05.

**Fig. 3:**
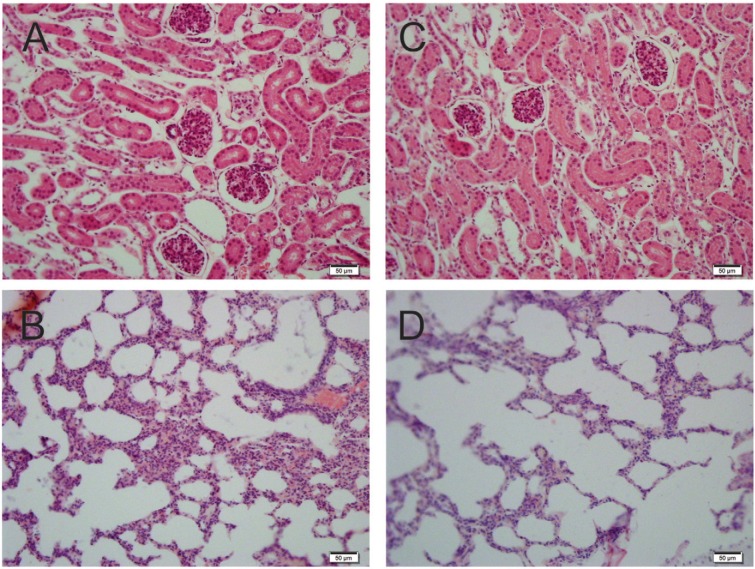
: histopathology of tissues stained with haematoxylin and eosin from hamsters that survived the lethal challenge. Kidneys (A, B) and lungs (C, D) from hamster immunised with recombinant fragment of LigBrep vaccine (DNA-prime/protein-boost and DNA/DNA + Alhydrogel, respectively) and challenged with virulent Leptospira interrogans. Note normal architecture observed in tissues (20X).

*Prophylactic effects of the vaccine preparations* - The protective efficacy of the vaccine preparations was monitored for up to 30 days post-challenge, in terms of survival, histopathological findings, and presence of*Leptospira* in the kidneys. The prime-boost strategy significantly protected 83.3% of animals (p < 0.05) (Fig. 2, Table), whereas 40% of hamsters immunised by the DNA/DNA vaccine-strategy survived (p < 0.05), presenting a median survival of 11 days. These animals presented negative kidney culture, as did the hamsters immunised with killed whole-leptospires, indicating the prophylactic effect of LigBrep when used under these immunisation strategies. Conversely, all animals that received the protein/protein or DNA/DNA vaccinations without adjuvant died during the experiment, presenting median survival times of 10 and nine days, respectively (Fig. 2). All hamsters of the positive control group, which were administered with killed whole-leptospires, were protected against mortality (p < 0.05), while those animals that received the empty pTARGET vector or PBS + Alhydrogel died (median survival = 8 days), confirming the high virulence of the challenge strain. No lesions were found in the organ samples collected from the surviving animals (Fig. 3). These findings indicate the capacity of these vaccines to induce sterilising immunity against leptospirosis.

**TABLE:** Immunoprotective efficacy of vaccine strategies using LigBrep

Vaccine	Surviving hamsters/ total of hamsters (n/n)	Death (endpoint days)	Survival (%)a
DNA-prime/protein-boost	5/6	11	83.3b
Protein/protein	0/6	8, 9, 10, 10, 11, 11	0
DNA/DNA (Alhydrogel)	2/5	11, 11, 11	40b
DNA/DNA (PBS)	0/5	8, 8, 9, 9, 10	0
pTARGET	0/5	8, 8, 8, 9, 9	0
PBS + Alhydrogel	0/5	8, 8, 8, 9, 9	0
Killed-whole leptospires	4/4	-	100b

*a*: surviving animals were observed for up to 30 days; *b*: p < 0.05 compared to negative control group [empty pTARGET or phosphate-buffered saline (PBS) + Alhydrogel].

## DISCUSSION

The development of an effective, safe, and cross-protective vaccine for the control of leptospirosis remains a challenge. Conserved antigens that are surface-exposed and possibly involved in pathogenesis have been evaluated as potential candidates for vaccine development (Ko et al. 2009,Dellagostin et al. 2011). Recently, we reported that the region shared by LigA and LigBrep, when presented as a DNA vaccine, protects hamsters against a heterologous challenge (Forster et al. 2013a). However, we believe that the efficacy of vaccines using LigBrep as antigen could be improved by other vaccination strategies. In this study, we evaluated DNA, protein, and prime-boost based vaccination, using LigBrep as antigen and Alhydrogel as adjuvant.

The functionality of the DNA vaccine vector was demonstrated in mammalian cells (VERO cells), and the IgG specific response in hamsters indicated that the antigen was successfully presented to immune system. DNA vaccines provide easy construction, efficient antigen delivery, induction of both humoral and cellular immunity, and a low cost of mass production, considering the lack of purification steps (Shams 2005). Additionally, some DNA vaccines are already licensed for veterinary use, while subunit vaccines are currently used in both humans and animals. Subunit vaccines are safe and elicit a primarily humoral immune response, however, the folding of the antigen produced under denaturing conditions can be impaired (Clark & Cassidy-Hanley 2005). Alternative expression systems are emerging, such as the yeast *Pichia pastoris*, which is already being used for leptospiral antigen production (Hartwig et al. 2010).

Vaccination protocols commonly require multiple immunisations to achieve a protective and sustained immune response. In particular, prime-boost vaccination with DNA vaccines and recombinant proteins has emerged as an effective strategy for eliciting a robust response against the target antigen. This strategy has been evaluated for the control of several diseases (Lu 2009), including leptospirosis (Hartwig et al. 2013). The fusion gene*lipL32-lipL41-ompL1* was evaluated using DNA, protein, and prime-boost strategies in BALB/c mice, although the mouse is not an adequate animal model for leptospirosis (Feng et al. 2009). Our group previously described the prime-boost strategy in a susceptible model using the LemA antigen (Hartwig et al. 2013). The efficacy of this protocol to induce protective immunity may depend on several factors such as the encoded antigens, animal species, and vaccine properties.

Notably, the prime-boost protocol, in which the DNA vaccine was firstly administered followed by a boost with rLigBrep, elicited protective immunity; more than 80% of animals vaccinated under the prime-boost protocol survived and showed sterilising immunity. We observed that the DNA, protein, and prime-boost vaccination methods induced different immune responses. Among the three immunisation strategies, the subunit vaccine using two doses of recombinant protein induced the highest IgG levels, but it was not protective. On the other hand, prime-boost vaccination induced lower IgG levels, but was able to protect hamsters from lethal challenge (83.3% protection). Previous studies have demonstrated that the modulation induced by DNA vaccines against leptospirosis was associated with a robust humoral immune response (Branger et al. 2005, Faisal et al. 2008, He et al. 2008), but there was no association between survival and IgG levels in our study. These results suggest a probable involvement of a Th1 mediated response. Studies have evaluated cytokine profiles induced by recombinant vaccines against leptospirosis (Faisal et al. 2008, 2009a, b, c, Yan et al. 2010); however, the mechanism underlying immune protection remains unknown.

Previous reports evaluated the protection against leptospirosis induced by LigBrep as recombinant subunit vaccine (aa 102-630) (Dellagostin et al. 2011). The evidences that this antigen is able to induce protection when administered as subunit vaccine are not very strong. In one of the studies that showed protection, the antigen was evaluated in mouse, a species not suitable as a model for leptospirosis (Koizumi & Watanabe 2004). In another study, a protection of 50-87% was achieved in the immunised group; however, 12-25% of the negative control group survived the challenge experiment (Yan et al. 2009). In our study, no significant protection in hamsters immunised with LigBrep as subunit vaccine was observed.

In this study, we included Alhydrogel, an adjuvant regularly used in commercial animal vaccines and approved for use in human vaccines (Petrovsky & Aguilar 2004), in vaccine formulations. The use of an adjuvant in DNA vaccines can increase their immunogenicity, and it has been reported that the use of aluminum as an adjuvant can increase protective efficacy and antibody titres by 10-100 fold, decreasing the dose of DNA vaccine required for immunisation by 10-fold (Kwissa et al. 2003). In the current study, we observed that the survival rates of hamsters immunised with DNA vaccine adsorbed on Alhydrogel were 40% higher than those of hamsters immunised with DNA vaccine without adjuvant. Additionally, DNA and prime-boost based-vaccines induced sterilising immunity in the surviving hamsters and reduced histopathological lesions. This is an important finding of our study, considering that sterilising immunity has only been reported once before, in a study where study hamsters were immunised with recombinant *Mycobacterium bovis* BCG expressing LipL32 (Seixas et al. 2007). Most vaccine candidates against leptospirosis have failed to induce sterilising immunity (Coutinho et al. 2011, Dellagostin et al. 2011, Zuerner et al. 2011).

In order to minimise the use of experimental animals, for ethical reasons, we performed only a single challenging experiment. That could be considered a weakness of the current study. However, the amount of data accumulated by our group after having performed a large number of similar experiments (Seixas et al. 2007, Silva et al. 2007, Felix et al. 2011, Forster et al. 2013a, Hartwig et al. 2013, 2014) provides confidence in our results.

In summary, we showed that LigBrep-based vaccination, especially with the prime-boost strategy, conferred high-level and sterilising protection against lethal infection in the hamster model of leptospirosis. These findings provide evidence that the conserved LigBrep region is a potential candidate antigen for the development of an effective and multivalent recombinant vaccine against leptospirosis.

## References

[B1] Adler B, Moctezuma AP (2010). Leptospira and leptospirosis. Vet Microbiol.

[B2] Babiuk LA, Babiuk SL, Loehr BI, Littel-van den Hurk SD (2000). Nucleic acid vaccines: research tool or commercial
reality. Vet Immunol Immunopathol.

[B3] Branger C, Chatrenet B, Gauvrit A, Aviat F, Aubert A, Bach JM, Andre-Fontaine G (2005). Protection against Leptospira interrogans sensu lato
challenge by DNA immunization with the gene encoding hemolysin-associated protein
1. Infect Immun.

[B4] Castiblanco-Valencia MM, Fraga TR, Silva LB, Monaris D, Abreu PA, Strobel S, Jozsi M, Isaac L, Barbosa AS (2012). Leptospiral immunoglobulin-like proteins interact with
human complement regulators factor H, FHL-1, FHR-1, and C4BP. J Infect Dis.

[B5] Chagas AD, McBride AJ, Athanazio DA, Figueira CP, Medeiros MA, Reis MG, Ko AI, McBride FW (2009). An imprint method for detecting leptospires in the
hamster model of vaccine-mediated immunity for leptospirosis. J Med Microbiol.

[B6] Choy HA (2012). Multiple activities of LigB potentiate virulence of
Leptospira interrogans: inhibition of alternative and classical pathways of
complement. PLoS ONE.

[B7] Choy HA, Kelley MM, Chen TL, Moller AK, Matsunaga J, Haake DA (2007). Physiological osmotic induction of Leptospira
interrogans adhesion: LigA and LigB bind extracellular matrix proteins and
fibrinogen. Infect Immun.

[B8] Clark TG, Cassidy-Hanley D (2005). Recombinant subunit vaccines: potentials and
constraints. Dev Biol.

[B9] Coutinho ML, Choy HA, Kelley MM, Matsunaga J, Babbitt JT, Lewis MS, Aleixo JAG, Haake DA (2011). A LigA three-domain region protects hamsters from lethal
infection by Leptospira interrogans. PLoS Negl Trop Dis.

[B10] Dellagostin OA, Grassmann AA, Hartwig DD, Felix SR, Silva EF, McBride AJ (2011). Recombinant vaccines against
leptospirosis. Hum Vaccin.

[B11] Faisal SM, Yan W, Chen CS, Palaniappan RU, McDonough SP, Chang YF (2008). Evaluation of protective immunity of Leptospira
immunoglobulin-like protein A (LigA) DNA vaccine against challenge in
hamsters. Vaccine.

[B12] Faisal SM, Yan W, McDonough SP, Chang CF, Pan MJ, Chang YF (2009a). Leptosome-entrapped leptospiral antigens conferred
significant higher levels of protection than those entrapped with PC-liposomes in
a hamster model. Vaccine.

[B13] Faisal SM, Yan W, McDonough SP, Chang YF (2009b). Leptospira immunoglobulin-like protein A variable region
(LigAvar) incorporated in liposomes and PLGA microspheres produces a robust immune
response correlating to protective immunity. Vaccine.

[B14] Faisal SM, Yan W, McDonough SP, Mohammed HO, Divers TJ, Chang YF (2009c). Immune response and prophylactic efficacy of smegmosomes
in a hamster model of leptospirosis. Vaccine.

[B15] Felix SR, Hartwig DD, Argondizzo AP, Silva EF, Seixas FK, Neto AC, Medeiros MA, Lilenbaum W, Dellagostin OA (2011). Subunit approach to evaluation of the immune protective
potential of leptospiral antigens. Clin Vaccine Immunol.

[B16] Feng CY, Li QT, Zhang XY, Dong K, Hu BY, Guo XK (2009). Immune strategies using single-component LipL32 and
multi-component recombinant LipL32-41-OmpL1 vaccines against
Leptospira. Braz J Med Biol Res.

[B17] Figueira CP, Croda J, Choy HA, Haake DA, Reis MG, Ko AI, Picardeau M (2011). Heterologous expression of pathogen-specific genes ligA
and ligB in the saprophyte Leptospira biflexa confers enhanced adhesion to
cultured cells and fibronectin. BMC Microbiol.

[B18] Forster KM, Hartwig DD, Seixas FK, Bacelo KL, Amaral M, Hartleben CP, Dellagostin OA (2013a). A conserved region of leptospiral immunoglobulin-like A
and B proteins as a DNA vaccine elicits a prophylactic immune response against
leptospirosis. Clin Vaccine Immunol.

[B19] Forster KM, Hartwig DD, Seixas FK, McBride AJ, Monte LG, Recuero AL, Brod CS, Hartleben CP, Amaral M, Dellagostin OA (2013b). Characterization of a virulent Leptospira interrogans
strain isolated from an abandoned swimming pool. Braz J Microbiol.

[B20] Gouveia EL, Metcalfe J, Carvalho AL, Aires TS, Villasboas-Bisneto JC, Queirroz A, Santos AC, Salgado K, Reis MG, Ko AI (2008). Leptospirosis-associated severe pulmonary hemorrhagic
syndrome, Salvador, Brazil. Emerg Infect Dis.

[B21] Hartskeerl RA, Collares-Pereira M, Ellis WA (2011). Emergence, control and re-emerging leptospirosis:
dynamics of infection in the changing world. Clin Microbiol Infect.

[B22] Hartwig DD, Bacelo KL, Oliveira PD, Oliveira TL, Seixas FK, Amaral MG, Hartleben CP, McBride AJ, Dellagostin OA (2014). Mannosylated LigANI produced in Pichia pastoris protects
hamsters against leptospirosis. Curr Microbiol.

[B23] Hartwig DD, Forster KM, Oliveira TL, Amaral M, McBride AJ, Dellagostin OA (2013). A prime-boost strategy using the novel vaccine
candidate, LemA, protects hamsters against leptospirosis. Clin Vaccine Immunol.

[B24] Hartwig DD, Oliveira TL, Seixas FK, Forster KM, Rizzi C, Hartleben CP, McBride AJ, Dellagostin OA (2010). High yield expression of leptospirosis vaccine
candidates LigA and LipL32 in the methylotrophic yeast Pichia
pastoris. Microb Cell Fact.

[B25] He HJ, Wang WY, Wu ZD, Lv ZY, Li J, Tan LZ (2008). Protection of guinea pigs against Leptospira interrogans
serovar Lai by LipL21 DNA vaccine. Cell Mol Immunol.

[B26] Ko AI, Goarant C, Picardeau M (2009). Leptospira: the dawn of the molecular genetics era for
an emerging zoonotic pathogen. Nat Rev Microbiol.

[B27] Koizumi N, Watanabe H (2004). Leptospiral immunoglobulin-like proteins elicit
protective immunity. Vaccine.

[B28] Koizumi N, Watanabe H (2005). Leptospirosis vaccines: past, present, and
future. J Postgrad Med.

[B29] Kwissa M, Lindblad EB, Schirmbeck R, Reimann J (2003). Codelivery of a DNA vaccine and a protein vaccine with
aluminum phosphate stimulates a potent and multivalent immune
response. J Mol Med.

[B30] Lin YP, Chang YF (2008). The C-terminal variable domain of LigB from Leptospira
mediates binding to fibronectin. J Vet Sci.

[B31] Lin YP, Lee DW, McDonough SP, Nicholson LK, Sharma Y, Chang YF (2009). Repeated domains of leptospira immunoglobulin-like
proteins interact with elastin and tropoelastin. J Biol Chem.

[B32] Lu S (2009). Heterologous prime-boost vaccination. Curr Opin Immunol.

[B33] Matsunaga J, Barocchi MA, Croda J, Young TA, Sanchez Y, Siqueira I, Bolin CA, Reis MG, Riley LW, Haake DA, Ko AI (2003). Pathogenic Leptospira species express surface-exposed
proteins belonging to the bacterial immunoglobulin superfamily. Mol Microbiol.

[B34] Matsunaga J, Sanchez Y, Xu X, Haake DA (2005). Osmolarity, a key environmental signal controlling
expression of leptospiral proteins LigA and LigB and the extracellular release of
LigA. Infect Immun.

[B35] McBride AJ, Cerqueira GM, Suchard MA, Moreira AN, Zuerner RL, Reis MG, Haake DA, Ko AI, Dellagostin OA (2009). Genetic diversity of the leptospiral immunoglobulin-like
(Lig) genes in pathogenic Leptospira spp. Infect Genet Evol.

[B36] Petrovsky N, Aguilar JC (2004). Vaccine adjuvants: current state and future
trends. Immunol Cell Biol.

[B37] Ramos CR, Abreu PA, Nascimento AL, Ho PL (2004). A high-copy T7 Escherichia coli expression vector for
the production of recombinant proteins with a minimal N-terminal His-tagged fusion
peptide. Braz J Med Biol Res.

[B38] Segura ER, Ganoza CA, Campos K, Ricaldi JN, Torres S, Silva H, Cespedes MJ, Matthias MA, Swancutt MA, Lopez Linan R, Gotuzzo E, Guerra H, Gilman RH, Vinetz JM (2005). Clinical spectrum of pulmonary involvement in
leptospirosis in a region of endemicity, with quantification of leptospiral
burden. Clin Infect Dis.

[B39] Seixas FK, Silva EF, Hartwig DD, Cerqueira GM, Amaral M, Fagundes MQ, Dossa RG, Dellagostin OA (2007). Recombinant Mycobacterium bovis BCG expressing the
LipL32 antigen of Leptospira interrogans protects hamsters from
challenge. Vaccine.

[B40] Shams H (2005). Recent developments in veterinary
vaccinology. Vet J.

[B41] Silva EF, Medeiros MA, McBride AJ, Matsunaga J, Esteves GS, Ramos JG, Santos CS, Croda J, Homma A, Dellagostin OA, Haake DA, Reis MG, Ko AI (2007). The terminal portion of leptospiral immunoglobulin-like
protein LigA confers protective immunity against lethal infection in the hamster
model of leptospirosis. Vaccine.

[B42] Yan W, Faisal SM, McDonough SP, Chang CF, Pan MJ, Akey B, Chang YF (2010). Identification and characterization of OmpA-like
proteins as novel vaccine candidates for leptospirosis. Vaccine.

[B43] Yan W, Faisal SM, McDonough SP, Divers TJ, Barr SC, Chang CF, Pan MJ, Chang YF (2009). Immunogenicity and protective efficacy of recombinant
Leptospira immunoglobulin-like protein B (rLigB) in a hamster challenge
model. Microbes Infect.

[B44] Zuerner RL, Alt DP, Palmer MV, Thacker TC, Olsen SC (2011). A Leptospira borgpetersenii serovar Hardjo vaccine
induces a Th1 response, activates NK cells, and reduces renal
colonization. Clin Vaccine Immunol.

